# A type II cannabis extract and a 1:1 blend of Δ(9)-tetrahydrocannabinol and cannabidiol display distinct antinociceptive profiles and engage different endocannabinoid targets when administered into the subarachnoid space

**DOI:** 10.3389/fphar.2023.1235255

**Published:** 2023-09-08

**Authors:** Besma Benredjem, Graciela Pineyro

**Affiliations:** ^1^ Département de Pharmacologie, Université de Montréal, Montreal, QC, Canada; ^2^ CHU Sainte-Justine Research Center, Montreal, QC, Canada

**Keywords:** phytocannabinoids, neuropathic pain, cannabinoid receptors, transient receptor potential vanilloid 1, mechanical allodynia, intrathecal injection

## Abstract

**Introduction:** Cannabis extracts are being increasingly used to mitigate chronic pain. Current guidelines for their prescription rely on Δ^9^-tetrahydrocannabinol (THC) and cannabidiol (CBD) content as well as the ratio of these major cannabinoids present in the blend. Here we assessed whether these descriptors were representative of product effectiveness to produce a desired outcome such as analgesia.

**Methods:** In this study, we used a rat model of diabetic neuropathy and assessed the reduction in mechanical allodynia following intrathecal injection of pure THC, pure CBD, a 1:1 mix of these compounds and a “balanced” chemotype II cannabis extract. Engagement of endocannabinoid targets by different treatments was investigated using CB1 (AM251) and CB2 (AM630) receptor antagonists as well as a TRPV1 channel blocker (capsazepine).

**Results:** Antinociceptive responses induced by an equivalent amount of THC administered in its pure form, as a THC:CBD mix or as a “balanced” extract were distinct. Furthermore, the 1:1 THC:CBD mix and the balanced extract had not only different response profiles but their relative engagement of CB1, CB2 receptors and TRPV1 channels was distinct.

**Discussion:** These findings indicate that antinociceptive responses and targets engaged by blended cannabinoids are composition-specific, and cannot be simply inferred from THC and CBD contents. This information may have implications in relation to the way medicinal cannabis products are prescribed.

## Introduction

Cannabis has been used for centuries for healing purposes (Russo, 2007), and its legalization has enhanced the widespread medicinal use of plant-based products (Park and Wu, 2017; Lintzeris et al., 2020). The plant produces more than 100 phytocannabinoids, of which Δ^9^-tetrahydrocannabinol (THC) and cannabidiol (CBD) are the most extensively studied for therapeutic purposes (Manzanares et al., 2006; Vuckovic et al., 2018). THC is also the main psychotropic component responsible for cannabis intoxication, whereas CBD lacks these effects and has been proposed to mitigate the undesirable psychoactive actions of THC (Leweke et al., 2012; Hudson et al., 2019). Based on such distinct profiles, the combined use of both cannabinoids is increasingly considered a viable strategy to benefit from the potential therapeutic properties of both agents while simultaneously mitigating the intoxicating effects of THC (MacCallum and Russo, 2018; Bhaskar et al., 2021; Bell et al., 2023).

The most common medicinal use of cannabis is for chronic pain management (Hill et al., 2017; Kosiba et al., 2019; Azcarate et al., 2020). Products that are used for this indication range from raw cannabis flowers to pharmaceutical-grade preparations. The latter include nabiximols, a balanced THC:CBD extract that is used in the management of pain and spasticity and that has been approved worldwide (Nurmikko et al., 2007; Collin et al., 2010; Russo et al., 2016; Bilbao and Spanagel, 2022). Although clinical evidence of the analgesic efficacy of cannabis products is, at best, moderate, their increasing use has prompted the development of prescription guidelines. According to the latter, established amounts of CBD and/or THC contained in commercial extracts of different strains are titrated to a maximum 1:1 combination to achieve relief (Bhaskar et al., 2021; Bell et al., 2023). Implicit in this practice is the notion that the specified doses of these cannabinoids will produce similar analgesia, independent of the composition of the product that is used for treatment. However, there is evidence that cannabinoids may interact with each other both at pharmacodynamic (Laprairie et al., 2015) and pharmacokinetic levels (Dumbraveanu et al., 2023; Zamarripa et al., 2023), raising the question as to whether analgesic equivalence between products of different compositions can be systematically assumed.

Cannabinoids elicit their analgesic effects via modulation of multiple pharmacodynamic targets, such as cannabinoid CB1 and CB2 receptors and TRPV1 (transient receptor potential vanilloid 1) channels (Ikeda et al., 2013; Morales et al., 2017; Muller et al., 2018; Zou and Kumar, 2018; Goncalves et al., 2022; Harding et al., 2023), all of which are upregulated in preclinical models of neuropathic pain (Lim et al., 2003; Hsieh et al., 2011; Vuckovic et al., 2018). Among chronic pain of neuropathic origin, diabetic neuropathy is one of the most common causes of hyperalgesia, hypoalgesia, and allodynia (Vinik et al., 2000; Aring et al., 2005). Such algetic manifestations and the upregulation of spinal CB1R, CB2R, and TRPV1 channels are present in streptozotocin-induced models of diabetic neuropathy (Hong and Wiley, 2005; Ikeda et al., 2013). In addition, intrathecal injection of synthetic cannabinoids in diabetic animals restores mechanical thresholds, implicating spinal and DRG targets of the endocannabinoid system in the analgesic effects of these ligands (Hong and Wiley, 2005; Ikeda et al., 2013; Goncalves et al., 2022). Here, we used this preclinical model of diabetic neuropathy to compare the antinociception induced by equivalent doses of THC administered in its pure form, as a 1:1 blend with pure CBD, or as a balanced THC:CBD extract. Administering the treatment in the subarachnoid space made it possible to avoid pharmacokinetic interactions associated with systemic administration of blended products (Dumbraveanu et al., 2023; Zamarripa et al., 2023). Consequently, we established that the antinociception induced by blended treatments of apparently equivalent composition not only differs in magnitude but also relies on distinct engagement of spinal/DRG targets of the endocannabinoid system.

## Materials and methods

### Animals

Adult male Sprague–Dawley (SD) rats, weighing 235–250 g, were purchased from Charles River Laboratories and housed in a controlled environment on a 12-h light/dark cycle with free access to food and water. All experimental methods and animal care procedures were approved by the Animal Care Committee of the University of Montreal (CDEA protocols 19-108 and 20-092) in accordance with the guiding principles as enunciated by the Canadian Council on Animal Care. Following experimentation, all rats were euthanized by CO_2_ asphyxiation and decapitation.

### Chemicals

WIN 55,212-2, Δ^9^-tetrahydrocannabinol (THC), cannabidiol (CBD), the cannabinoid 2 receptor antagonist AM630 (6-iodo-2-methyl-1-[2-(4-morpholinyl)ethyl]-1*H*-indol-3-yl](4-methoxyphenyl) methanone), the cannabinoid 1 receptor antagonist AM251 (*N*-(piperidin-1-yl)-5-(4-iodophenyl)-1-(2,4-dichlorophenyl)-4-methyl-1*H*-pyrazole-3-carboxamide), and the TRPV1 antagonist capsazepine were purchased from Cayman Chemical (Burlington, Canada). Stock solutions of the drugs were prepared in dimethyl sulfoxide (DMSO) for WIN 55,212-2 (25 mM); in ethanol 100% for CBD (100 mM), THC (100 mM), AM251 (18 mM), and capsazepine (26 mM); and in dimethylformamide (DMF) for AM630 (20 mM). The cannabis extract used in this study was a kind gift from Canopy Growth (Smiths Falls, Ontario, Canada) and was provided in a stock solution of 100% ethanol containing THC at a 230 mM concentration. The percent w/w content of THC and CBD was 38% and 55% (1:1.4 ratio), respectively, corresponding to a chemotaxonomy type II profile where THC and CBD are considered “balanced” (Wallach, 2021). The extract also contained small amounts of cannabinol (CBN), cannabichromene (CBC), and cannabigerol (CBG) with w/w contents of 1.50, 3.27, and 1.24%, respectively. The extract used did not contain volatile terpenes or flavonoids. Streptozotocin (STZ) was purchased from Cayman Chemical (Burlington, Canada) and dissolved in citrate buffer (10 mM, pH 4.5).

### Induction of diabetic neuropathic pain and assessment of mechanical allodynia

STZ-induced diabetes displays sensory abnormalities that mimic human neuropathy, such as mechanical allodynia (Talbot and Couture, 2012), and the model responds to cannabinoids (Vera et al., 2012; Ikeda et al., 2013; Goncalves et al., 2022). Hence, streptozotocin (STZ) was administered systemically at 60 mg kg^−1^, i.p. to allow for the development of persistent diabetic neuropathy (Talbot et al., 2010; Bagheri Tudashki et al., 2020). One week after STZ injection, the development of hyperglycemia was confirmed using an Accu-Chek Aviva glucometer (Roche Diagnostics) to measure glucose levels in blood samples taken from the tail vein. Rats with blood glucose levels between 20 and 28 mmol/L were considered diabetic (Talbot and Couture, 2012; Othman et al., 2019). Rats injected with the citrate buffer vehicle were used as controls.

In the second week following STZ injection, mechanical allodynia was evaluated using von Frey filaments as previously described (Charfi et al., 2018; Bagheri Tudashki et al., 2020). Briefly, the rats were accustomed for 15 min on a metal mesh floor under an inverted plastic box (20 × 10 × 10 cm) in a quiet room. After habituation, the plantar surfaces of the right and left hind paws were alternately touched (6–8 s) by applying calibrated filaments from underneath the cage through openings in the mesh floor to the hind paw. A series of von Frey filaments of progressively wider diameter (4, 6, 8, 10, 15, and 26 g) were used to determine the threshold of pressure required to produce withdrawal. Brisk withdrawal or paw flinching was considered a positive response. In the absence of a response at a pressure of 26 g, animals were assigned this cutoff value. The mechanical threshold response was obtained by consigning the pressure in grams, which would result in withdrawal of the paw in 50% of 10 trials (Charfi et al., 2018; Bagheri Tudashki et al., 2020). Von Frey measurements were taken every 15 min for 120 min after administration of active cannabinoids.

### Treatment groups

#### Cannabinoid analgesia

In the first series of experiments, we examined dose-dependent analgesic responses to different cannabinoids. For this purpose, animals were divided into five experimental groups. Measures were then taken in animals that received intrathecal (i.t.) injections of either vehicle or increasing doses of the following drugs: Group #1 WIN 55,212-2 (0.2–60 nmoles, five doses); Group #2 THC (95–725 nmoles, four doses); Group #3 CBD (95–725 nmoles, four doses); Group #4 THC:CBD (1:1) mixture (four doses); and Group #5 cannabis extract (four doses). When the THC:CBD (1:1) mixture or the cannabis extract was injected, it delivered equivalent amounts of THC as when this cannabinoid was administered in its pure form. Within each treatment group, the rats were randomized to receive vehicle or different doses at the beginning of each week, and treatments were codified so that the experimenter was blind to experimental conditions during behavioral testing. Each rat received only one treatment and a single dose per day. If animals received more than one treatment, they were allowed to recover for 7 days before an injection was repeated. The total number of animals per treatment group was as follows: Group #1: 19 rats; Group #2: 16 rats; Group #3: 16 rats; Group #4: 16 rats; and Group #5: 17 rats. Each drug dose or corresponding vehicle was assessed independently five times (*n* = 5). The doses of WIN 55,212-2 that were used were based on published studies (Fox et al., 2001; Rahn et al., 2007; Brownjohn and Ashton, 2012). The doses of THC administered in its different forms were based on a pilot study in which we evaluated the analgesic effects of pure THC at seven different doses ranging from 95 to 955 nmoles, four of which were retained for the study. Vehicles that were used with different treatments, and their lack of effect on baseline values is shown in Supplementary Tables S1, S2.

#### Antagonism experiments

In a second series of experiments, the CB1R antagonist AM251 (10–20 nmoles), the CB2R antagonist AM630 (10–20 nmoles), or the TRPV1 channel blocker capsazepine (12.5–50 nmoles) were administered (i.t.) 30 min prior to the delivery of the active treatments: Group #1 WIN 55,212-2 (20 nmoles); Group #2 THC (250 nmoles); Group #3 extract (250 nmoles THC equivalent); Group #4 THC (480 nmoles); Group #5 extract (480 nmoles THC equivalent); Group #6 THC:CBD (1:1) mix (480 nmoles THC equivalent); and Group #7 CBD (725 nmoles). The composition of the vehicles used to deliver these different treatments is shown in Supplementary Table S3, none of which had an effect on paw withdrawal thresholds (Supplementary Table S4). The doses of the CB1R, CB2R, and TRPV1 antagonists used in the study were based on the literature (Kanai et al., 2006; Romero-Sandoval and Eisenach, 2007; Ueda et al., 2014) and were chosen following a pilot study in which we ensured that, when administered alone, these drugs did not modify basal withdrawal thresholds in neuropathic animals (Supplementary Figure S1). The rats were randomized to receive different antagonist + treatment combinations at the beginning of each week, and treatments were codified so that the experimenter was blind to conditions during behavioral testing. Each rat received only one treatment combination per day. If animals received more than one treatment, they were allowed to recover for 7 days before an injection was repeated. Each antagonist/agonist combination was independently assessed six times (*n* = 6). The total number of animals per treatment group was as follows: Group #1: 16 rats; Group #2: 19 rats; Group #3: 17 rats; Group #4: 22 rats; Group #5: 21 rats; Group #6: 21 rats; and Group #7: 20 rats.

### Intrathecal injections

Injections were carried out as previously described (Bagheri Tudashki et al., 2020). Briefly, rats were put under light anesthesia using 4% isoflurane inhalation in a 1:1 mixture of oxygen and air until a loss of the righting reflex was observed (approximately 3 min). The rats were then shaved on the lower back to help visualize the lumbar region and placed in a nose cone for continued isoflurane administration during the procedure. A 27G × ½ needle attached to a 50-μL Hamilton syringe was inserted at the mid-lumbar level into the subarachnoid space. A quick movement of the tail indicated that the needle had entered the subarachnoid space, and the solution was slowly injected (30 μL for analgesic treatments; 20 μL for antagonists). The animals were allowed to recover for 15 min before mechanical thresholds were determined. Treatments were administered intrathecally to avoid pharmacodynamic interactions that could modify the bioavailability of blended cannabinoids when they were administered systemically. Doing so allowed for better control of the effective concentration of cannabinoids interacting with their intended pharmacodynamic targets accessible from the subarachnoid space.

### Data analysis

Following the acquisition of the time course of withdrawal thresholds, these responses were integrated over 120 min. Since analgesia by different treatments and different doses had distinct time courses, integration over this fixed time period ensured the termination of analgesic effects for all treatments, thus allowing for comparisons between them. The values obtained through the integration of thresholds over time were consigned as the area under the curve (AUC). All statistical comparisons were performed using GraphPad 7 (GraphPad Software). The evolution of weight gain and glycemia in rats injected with STZ or citrate buffer was evaluated using a two-way ANOVA followed by Sidak’s or Tukey’s *post hoc* test, as indicated in the corresponding figures. A comparison of mechanical withdrawal thresholds obtained in rats injected with STZ vs. citrate buffer was evaluated by Student’s t-test. The time course of withdrawal thresholds for different doses of each treatment was analyzed by a two-way ANOVA followed by Tukey’s *post hoc* test. AUC values generated for the different doses of each cannabinoid were compared to the AUC values of the corresponding vehicles using a one-way ANOVA followed by Sidak’s *post hoc* tests. Statistical analysis of the effects of CB1R, CB2R, or TRPV1 antagonists on the antinociception elicited by different cannabinoids was performed by a one-way ANOVA followed by Dunnett’s *post hoc* test.

## Results

### Mechanical allodynia in STZ-injected rats

Assessment of the evolution of glycemia and body weight following injection of STZ or vehicle (citrate buffer) confirmed the development of diabetes in STZ-injected rats. Analysis of glycemia by a two-way ANOVA showed an effect of STZ treatment *F* (1,30) = 291.10 (*p* < 0.0001), an effect of time *F* (2,30) = 77.41 (*p* < 0.0001), and an interaction *F* (2,30) = 64.98 (*p* < 0.0001). Tukey’s *post hoc* test revealed that STZ-injected but not citrate-injected rats displayed a significant increase in glucose levels 1 and 2 weeks after STZ injection (*p* < 0.0001; Supplementary Figure S2A), indicating the development of diabetes in the former but not in the latter. Similar analysis of body weight gain indicated an effect of treatment F (1, 30) = 13.23 (*p* = 0.001), an effect of time F (2, 30) = 86.68 (*p* < 0.0001), and an interaction F (2, 30) = 14.62 (*p* < 0.0001). Sidak’s *post hoc* test indicated that the STZ group failed to gain weight during the second week after injection (*p* = 0.65; Supplementary Figure S2B), a feature that is typical of type I diabetes (Goncalves et al., 2022). A comparison using Student’s t*-*test indicated that the mean withdrawal thresholds in STZ rats (3.36 ± 0.15; *n* = 6) were significantly lower than those of rats injected with citrate buffer (17.62 ± 3.7; *n* = 6; *p* = 0.0036), thus confirming the presence of mechanical allodynia in diabetic animals.

### Pure cannabinoids induce antinociceptive responses

**FIGURE 1:**
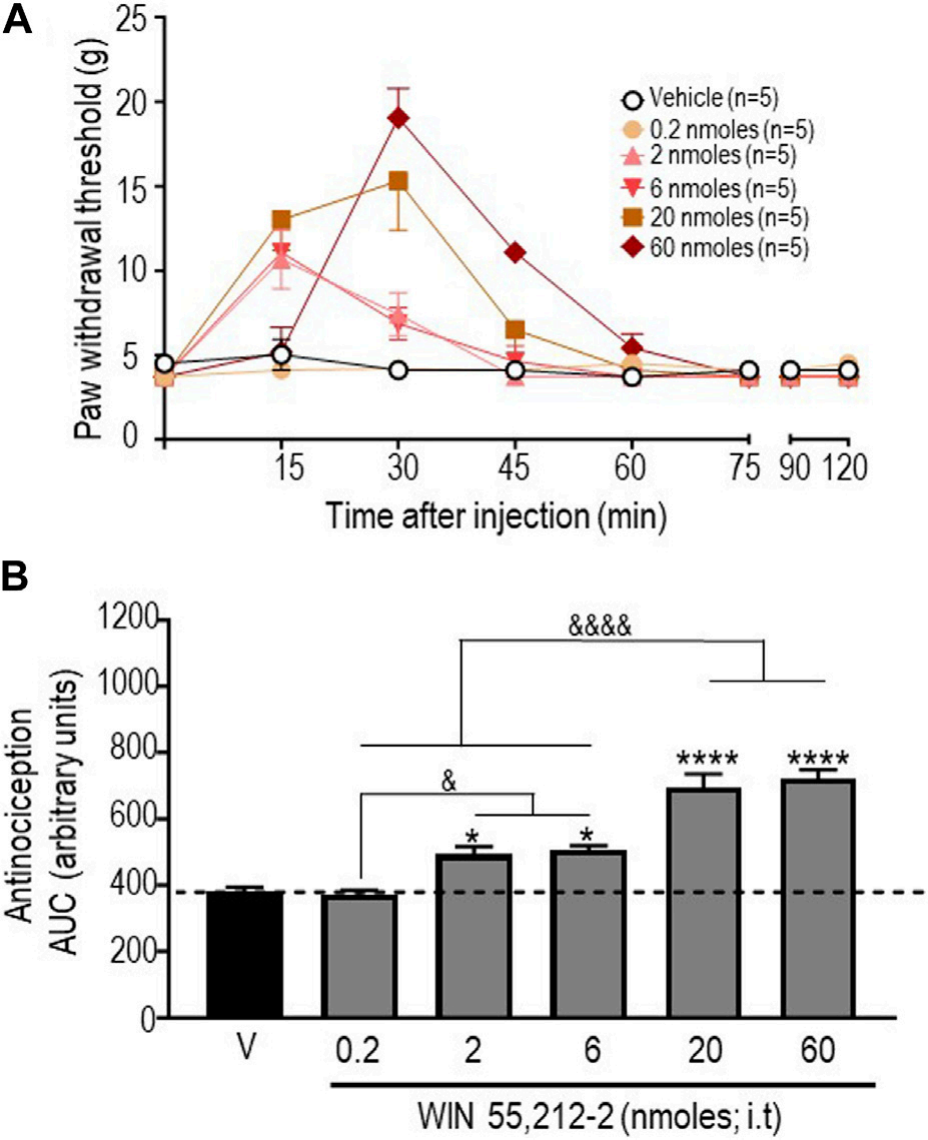
Anti-allodynic effects of WIN 55, 212-2 in STZ rats. One week after administration of STZ, rats received i.t. injections of WIN 55, 212-2 at the indicated doses. **(A)** Mechanical thresholds were assessed every 15 min after injection for a period of 120 min, thus allowing all responses to return to baseline. **(B)** Histograms show AUC (mean ± SEM; *n* = 5) corresponding to withdrawal thresholds integrated over 120 min and are expressed in arbitrary units. A one-way ANOVA followed by Sidak’s multiple comparison test was used to compare anti-allodynic responses. *p < 0.05 and ****p < 0. 0001 compared to vehicle; & *p* < 0.05 and &&&& *p* < 0.0001, as indicated.

In an initial series of experiments, we used the CB1R/CB2R agonist WIN 55,212-2 (Felder et al., 1995) to verify that responses to this reference compound were comparable to those previously described in the STZ rat model (Ikeda et al., 2013). The time courses of mechanical withdrawal thresholds elicited by increasing doses of this cannabinoid are shown in Figure 1A. A two-way ANOVA analysis of the data indicated an effect of time F (7, 192) = 43.65 (*p* < 0.0001), an effect of dose F (5, 192) = 13,88 (*p* < 0.0001), and an interaction F (35, 192) = 9,931 (*p* < 0.0001). Tukey’s *post hoc* test further indicated that the maximum withdrawal threshold (19.04 ± 1.74 g) was attained following administration of 60 nmoles of this ligand (*p* < 0.0001). Withdrawal responses integrated over time (AUC) for increasing doses of this agonist are shown in Figure 1B. A one-way ANOVA of AUC values confirmed that WIN 55,212-2 was antinociceptive (*p* < 0.0001), and subsequent comparisons using Sidak’s *post hoc* test revealed significant antinociception starting at 2 nmoles (*p* = 0.0175 for 2 moles; *p* = 0.0082 for 6 nmoles; *p* < 0.0001 for 20 nmoles; and *p* < 0.0001 for 60 nmoles). The time course and dose range of the observed responses are consistent with previous effects reported for i.t. injection of WIN 55,212-2 in STZ (Ikeda et al., 2013) and other neuropathic pain models (Brownjohn and Ashton, 2012; Ikeda et al., 2013).

**FIGURE 2:**
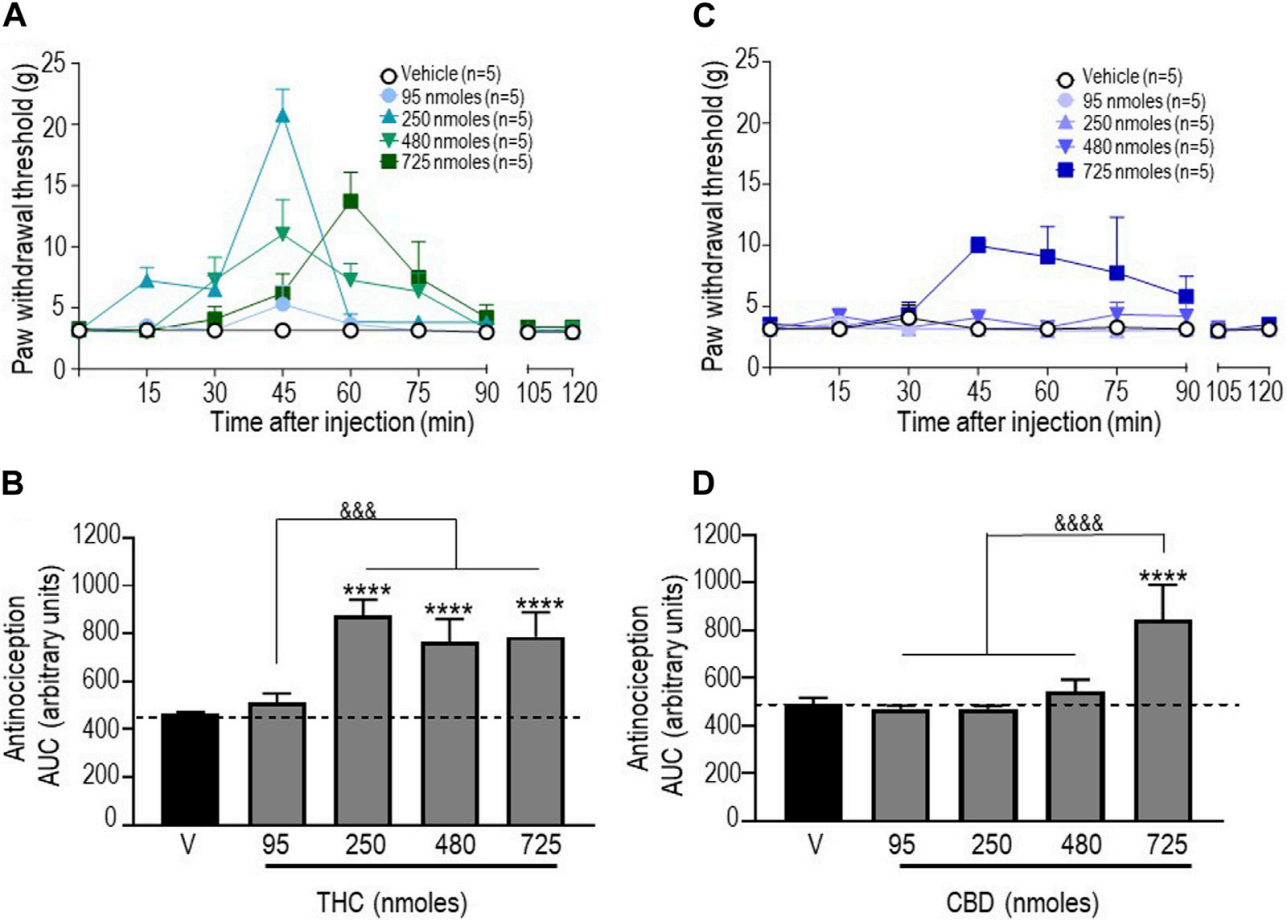
Anti-allodynic effects of THC and CBD in STZ rats. One week after STZ administration, rats received i.t. injections of **(A)** THC or **(C)** CBD at the indicated doses. Mechanical thresholds were assessed every 15 min after injection for a period of 120 min, thus allowing all responses to return to baseline. **(B, D)** Histograms show AUC values (mean ± SEM; *n* = 5) corresponding to withdrawal thresholds integrated over 120 min and are expressed in arbitrary units. A one-way ANOVA followed by Sidak’s multiple comparison test was used to compare anti-allodynic responses. ****p < 0.0001 compared to vehicle; &&&& *p* < 0.0001, comparison as indicated.

Next, antinociceptive responses following administration of pure THC were assessed. Since vehicles for the different doses of this cannabinoid that were tested had no effect (Supplementary Table S2), withdrawal thresholds obtained with vehicles were pooled to produce baseline withdrawal values, as shown in Figure 2A. A two-way ANOVA analysis of mechanical withdrawal thresholds indicated an effect of time F (8, 180) = 8.810 (*p* < 0.0001), an effect of dose F (4, 180) = 9.462 (*p* < 0.0001), and an interaction F (32, 180) = 3.485 (*p* < 0.0001), while Tukey’s *post hoc* tests revealed the maximum threshold of 20.78 ± 2.13 g attained 45 min after administration of 250 nmoles (*p* < 0.0001). AUC values for each dose (Figure 2B) were then compared using a one-way ANOVA (*p* < 0.0001), with Sidak’s *post hoc* test indicating that the antinociceptive responses integrated over time were significant at 250 nmoles (*p* < 0.0001), with no further increase at higher THC doses (*p* < 0.0001 for 480 nmoles; *p* < 0.0001 for 725 nmoles).

Similar analysis of withdrawal thresholds elicited by different doses of CBD indicated a significant effect of time F (8, 180) = 2.099 (*p* = 0.0380), dose F (4, 180) = 12.22 (*p* < 0.0001), and an interaction F (32, 180) = 1.856 (*p* = 0.0062) with a maximum threshold of 9.97 ± 0.68 g attained 45 min after administration of the highest dose (725 nmoles; *p* < 0.001). As shown in Figure 2D, the only difference between AUC values for different CBD doses and vehicle is the time-integrated response to 725 nmoles (one-way ANOVA *p* < 0.0001; Sidak’s test *p* < 0.0001).

### Administration of a 1:1 mixture of pure THC:CBD and a “balanced” (type II) extract induces antinociceptive responses

**FIGURE 3:**
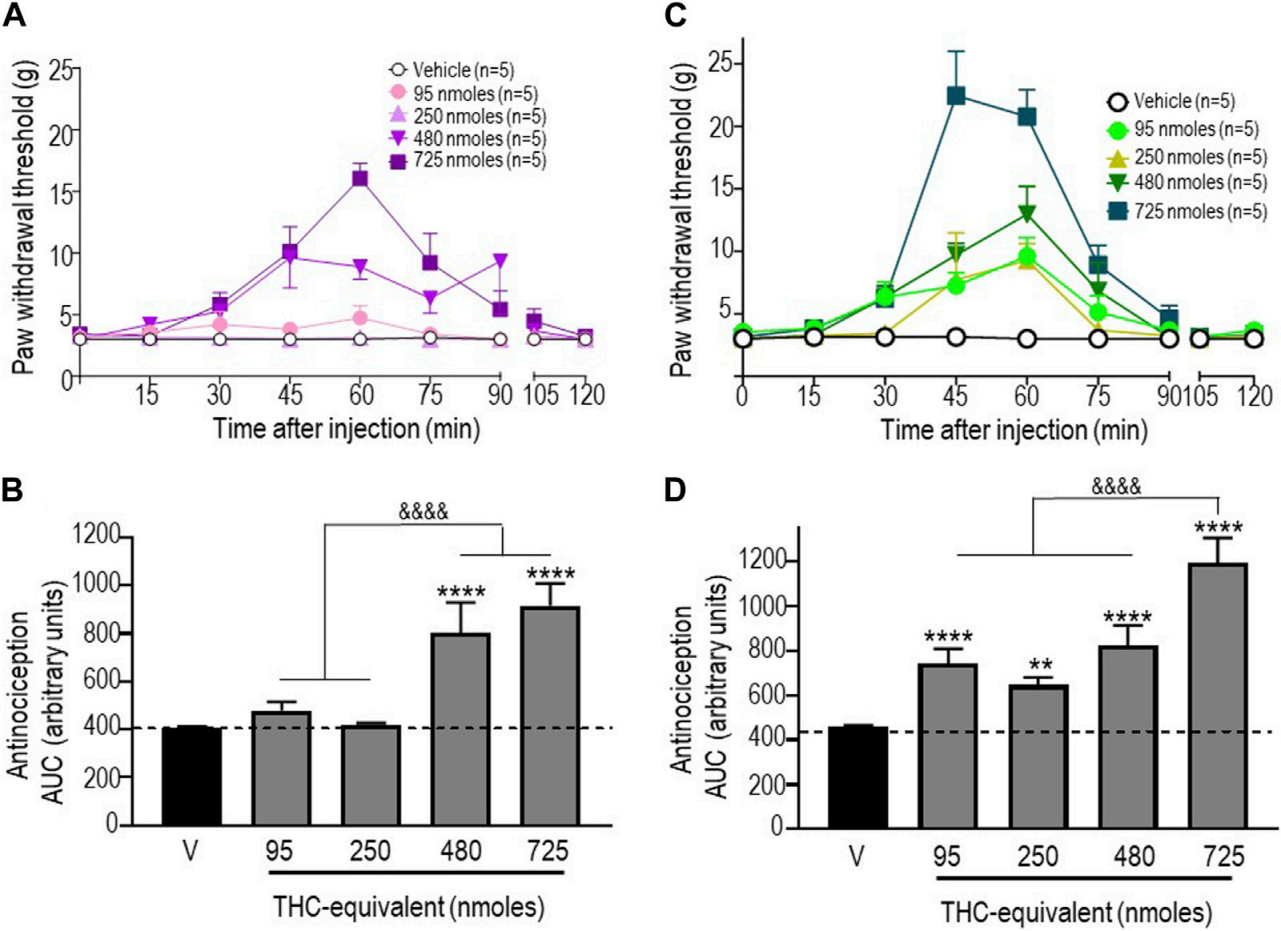
Anti-allodynic effects of the THC:CBD (1:1) mixture and the extract in STZ rats. One week after STZ administration, rats received i.t. injections of **(A)** the THC:CBD (1:1) mixture or **(C)** the cannabis extract at the indicated THC-equivalent doses. Mechanical thresholds were assessed every 15 min after injection for a period of 120 min, thus allowing all responses to return to baseline. **(B, D)** Histograms show AUC values (mean ± SEM; *n* = 5) corresponding to withdrawal thresholds integrated over 120 min and are expressed in arbitrary units. A one-way ANOVA followed by Sidak’s multiple comparison test was used to compare anti-allodynic responses. **p < 0.01 and ****p < 0.0001 compared to vehicle; & *p* < 0.05 and &&&& *p* < 0.0001, as indicated.

We next examined the antinociceptive responses of a 1:1 mixture of pure THC:CBD and of a “balanced” extract, both of which were administered so that the doses of THC injected with these treatments were equivalent to those used when THC was injected in its pure form. As mentioned previously, the baseline values shown in Figure 3 correspond to pooled vehicle responses, which were ineffective by themselves (Supplementary Table S2). The time courses of mechanical withdrawal thresholds for increasing concentrations of THC administered as a mix with pure CBD at a ratio of 1:1 are shown in Figure 3A. Analysis by a two-way ANOVA revealed an effect of time F (8, 180) = 8.810 (*p* < 0.0001), an effect of dose F (4, 180) = 9.462 (*p* < 0.0001), and an interaction F (32, 180) = 3.485 (*p* < 0.0001), with a maximum threshold of 16.06 ± 1.24 g attained 45 min after administration of 750 nmol-equivalent THC (*p* < 0.001; Tukey’s test). A comparison of AUC values for withdrawal responses integrated over time was performed by a one-way ANOVA, which revealed an overall effect of treatment (*p* < 0.0001), while Sidak’s *post hoc* comparisons indicated that only the 480 and 725 nmoles produced significant antinociceptive responses (*p* < 0.0001), without a significant difference between the two doses (*p* = 0.099; Figure 3B).

Similar analyses were completed when THC was given as an extract. A two-way ANOVA of the time course of mechanical withdrawal thresholds indicated an effect of time F (8, 180) = 30.11 (*p* < 0.0001), an effect of dose F (4, 180) = 23.38 (*p* < 0.0001), and an interaction F (32, 180) = 5.570 (*p* < 0.0001). The maximal response induced by this treatment was 22.46 ± 3.54 g, which was attained 45 min after the administration of 750 nmol-equivalent THC (*p* < 0.0001 Tukey’s test; Figure 3C). A comparison of AUC values by a one-way ANOVA also revealed a significant effect of treatment at different doses tested (*p* < 0.0001; *p* < 0.0001 for 95 nmoles; *p* = 0.0054 for 250 nmoles; *p* < 0.0001 for 480 nmoles; and *p* < 0.0001 for 725 nmoles).

### The antinociceptive profile of THC differs when administered as a pure cannabinoid, as part of a 1:1 THC:CBD mixture, or as a “balanced” (type II) extract

**FIGURE 4:**
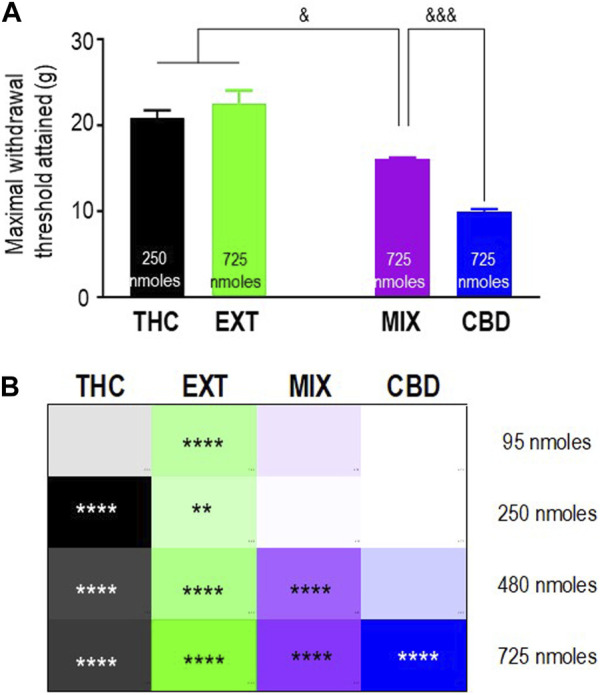
Comparison of the anti-allodynic effects of THC, CBD, THC:CBD (1:1) mix, and extract in STZ rats. Histograms show maximum withdrawal thresholds (mean ± SEM, *n* = 5) elicited by different treatments; doses at which maximal response was observed are indicated. **(A)** Maximal responses were compared by a one-way ANOVA followed by Sidak’s multiple comparisons to reveal differences between different treatments. & *p* < 0.05 and &&& *p* < 0.001, as indicated. Graphic summary of time-integrated responses elicited by increasing doses of the indicated treatments. **(B)** AUC values in Figures 2, 3 are presented here as a heatmap, along with the corresponding statistics in the figures.

To verify the extent to which “entourage” cannabinoids modified the antinociceptive responses induced by THC, we then sought to compare mechanical allodynia in diabetic animals that received this cannabinoid in its pure form, as part of a 1:1 mix with pure CBD, or as a component within the “balanced” (type II) cannabis extract. Pure CBD was also included in the comparison to determine if the combination with THC had an influence on CBD responses. The maximum mechanical thresholds attained by the different treatments are shown in Figure 4A, and their analysis by one-way ANOVA revealed overall differences between treatments (*p* < 0.0001). Sidak’s *post hoc* test further indicated that the maximal responses attained by 250 nmoles of pure THC and 725 nmoles of the extract were similar to one another (*p* = 0.6497) but higher than the maximum threshold attained by 725 nmoles of the mix (*p* < 0.05). In turn, the maximal response induced by the mix was higher than that elicited by 725 nmoles of CBD (*p* < 0.001). To facilitate the comparison of the time-integrated responses (AUC values), the results from the previous sections (Figures 2B, D; Figures 3B, D) are summarized as a heatmap as shown in Figure 4B. When considering AUC values, an effective antinociceptive effect was evident when 95 nmol-equivalent THC was administered as an extract but not as a mix or in its pure form. At 250 nmoles, THC was effective when given as an extract or in its pure form but not when administered as a 1:1 mix with CBD. It was only at 480 nmol-equivalent THC that all products containing this cannabinoid produced effective antinociception. On the other hand, a dose of 725 nmoles of pure CBD was required to significantly modify AUC values beyond those observed in vehicle-treated animals.

### Contributions of CB1/CB2 receptors and TRPV1 channels to analgesia induced by different cannabinoids

CB1R, CB2R, and TRPV1 channels are upregulated in the spinal cord and the DRGs of different models of neuropathic pain (Lim et al., 2003; Hsieh et al., 2011; Vuckovic et al., 2018), such as diabetic neuropathy (Hong and Wiley, 2005; Ikeda et al., 2013). Moreover, considerable evidence links these membrane targets of the endocannabinoid system to the analgesic effects of cannabinoids (Costa et al., 2004; Ikeda et al., 2013; Milligan et al., 2020). Hence, to further explore similarities and differences in the antinociceptive responses induced by pure and blended cannabinoids, we investigated whether these different targets were distinctively engaged by the different treatments. To this end, we compared anti-allodynic responses induced by the different cannabinoids in the absence and presence of either the CB1R antagonist AM251, the CB2R antagonist AM630, or the TRPV1 channel blocker capsazepine (Kanai et al., 2006; Romero-Sandoval and Eisenach, 2007; Ueda et al., 2014). At the doses used, none of the antagonists had an effect by themselves (Supplementary Figure S1); however, as described in the following paragraphs, they distinctively interfered with the antinociception induced by different cannabinoids.

**FIGURE 5:**
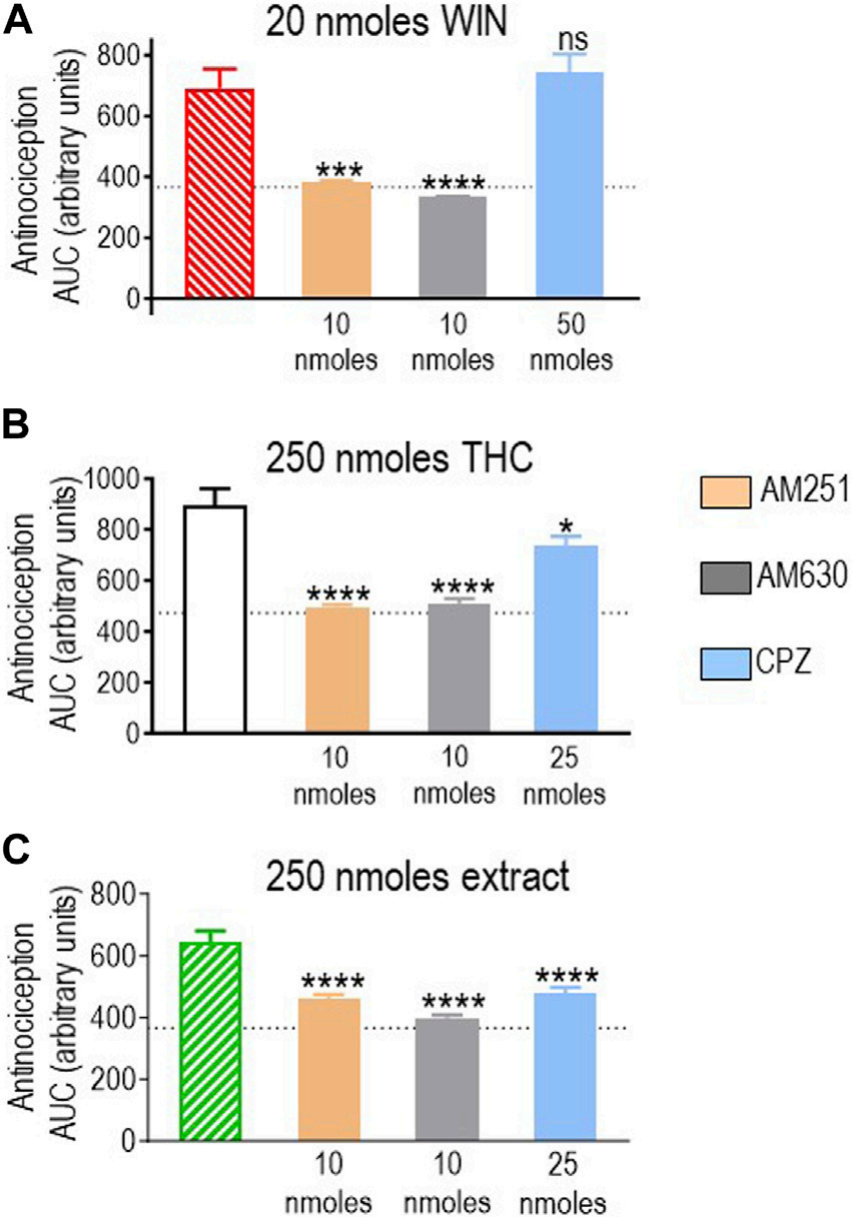
Effects of CB1R/CB2R antagonists and TRPV1 channel blockers on the anti-allodynic effects induced by WIN 55,212-2 (20 nmoles), pure THC, and the type II extract (250 nmoles). One week after induction of diabetes by STZ and 30 min before i.t. administration of **(A)** WIN 55,212-2, **(B)** THC, or **(C)** the balanced extract, rats were pre-injected (i.t.) with the selective CB1R antagonist AM251 (orange), the selective CB2R antagonist AM630 (gray), or the selective TRPV1 channel blocker capsazepine (sky blue bar), at the doses indicated in the figure. Histograms show AUC values of withdrawal thresholds integrated over 120 min and are expressed in arbitrary units. They correspond to the mean ± SEM, *n* = 6. Statistical comparisons were carried out using a one-way ANOVA followed by Dunnett’s multiple comparison test. *p < 0.05, ***p < 0.001, and ****p < 0.0001 comparing antagonist vs. no antagonist pretreatment. Dotted lines indicate AUC values obtained in vehicle-injected animals, as detailed in the Materials and Methods section.

First, we assessed how antagonists influenced the response elicited by 20 nmoles of the reference ligand WIN 55,212-2, and the results are shown in Figure 5A. A one-way ANOVA analysis revealed an effect of antagonists (*p* < 0.0001), and the subsequent use of Dunnett’s test to compare AUC values obtained in the presence vs. the absence of antagonists indicated that blockade of CB1R by AM251 (10 nmoles; *p* < 0.001) and CB2R by AM630 (10 nmoles; *p* < 0.0001) abolished WIN 55,212-2-mediated antinociception, resulting in corresponding AUC values to those observed in animals that received the vehicle. In contrast, 50 nmoles of capsazepine, which effectively abolished the response to the highest doses of other cannabinoids, did not show an effect on WIN 55,212-2 (*p* = 0.7619; Figure 5A), confirming this synthetic cannabinoid as a reference for CB1R/CB2R-mediated antinociception in the diabetic neuropathy model used here.

We next evaluated the effect of different antagonists on responses elicited by pure or blended THC. We had identified 250 nmoles as the lowest effective dose of pure THC. This dose effectively induced antinociception when administered as a balanced extract but not as a 1:1 mix with pure CBD (Figure 4B). Hence, we compared how CB1R and CB2R antagonists, in addition to TRPV1 blockers, influenced the effective responses elicited by 250 nmoles of THC in the products in which significant activity had been previously observed. A one-way ANOVA confirmed an effect of antagonist pretreatment both on AUC values obtained with pure THC (*p* < 0.0001; Figure 5B) and the extract (*p* = 0.0002; Figure 5C). Dunnett’s *post hoc* comparisons indicated that the CB1R antagonist AM251 (10 nmoles) abolished THC antinociception (*p* < 0.0001) and reduced that of the extract, leading to an increase of ∼26% higher than vehicle values (*p* < 0.0001). The CB2R antagonist AM630 suppressed antinociception by both treatments (*p* < 0.0001), while the TRPV1 antagonist capsazepine (25 nmoles) was also active, partially reducing analgesia by THC (*p* = 0.0151) and the extract (*p* < 0.0001). Thus, analgesia elicited by 250 nmoles of THC administered in its pure form or as an extract resembled the synthetic cannabinoid WIN 55,212-2 in its ability to engage CB1R/CB2R. Additionally, both THC-containing treatments were sensitive to TRPV1-blockade by capsazepine, which was not the case for WIN 55,212-2.

**FIGURE 6:**
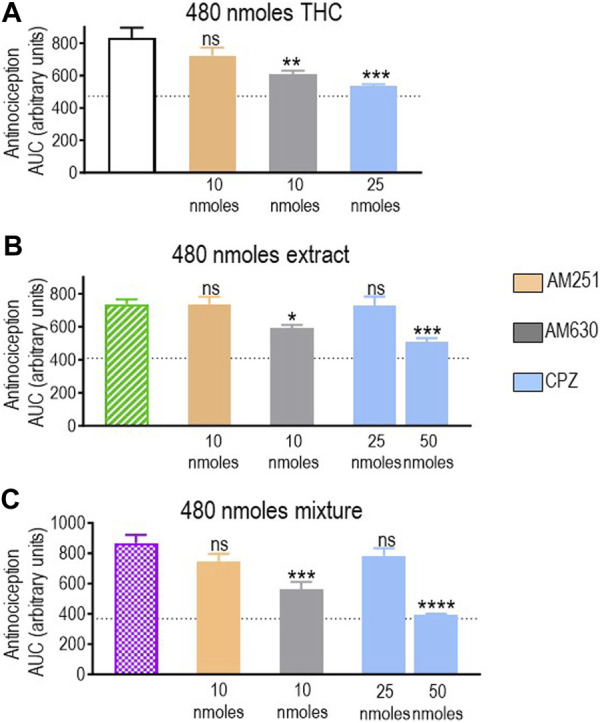
Effects of CB1R/CB2R antagonists and TRPV1 channel blockers on the anti-allodynic effects induced by THC administered in its pure form, as a 1:1 THC:CBD mix, or as the type II extract (480 nmoles). One week after induction of diabetes by STZ and 30 min before i.t. administration of **(A)** pure THC, **(B)** the balanced extract THC, or **(C)** the 1:1 THC:CBD mix, rats were pre-injected (i.t.) with the selective CB1R antagonist AM251 (orange), the selective CB2R antagonist AM630 (gray), or the selective TRPV1 channel blocker capsazepine (sky blue bar), at the doses indicated in the figure. Histograms show AUC values of withdrawal thresholds integrated over 120 min and are expressed in arbitrary units. They correspond to mean ± SEM, *n* = 6. Statistical comparisons were carried out using a one-way ANOVA followed by Dunnett’s multiple comparison test. *p < 0.05, **p < 0.01, ***p < 0.001, and ****p < 0.0001 comparing antagonist vs. no antagonist pretreatment. Dotted lines indicate AUC values obtained in vehicle-injected animals, as detailed in the Materials and Methods section.

Pure THC, the extract, and the THC:CBD 1:1 mix all elicited significant antinociception when tested at a dose of 480 nmol-equivalent THC (summarized in Figure 4B). Therefore, we next evaluated whether and how CB1R, CB2R, and TRPV1 blockers interfered with responses elicited by this dose of THC in different preparations. A one-way ANOVA confirmed an effect of antagonist pretreatment for pure THC (*p* < 0.001; Figure 6A), extract (*p* < 0.001; Figure 6B), and 1:1 mix (*p* < 0.0001; Figure 6C). Corresponding Dunnett’s tests indicated that the CB1R antagonist AM251 (10 nmoles) did not block responses by this higher dose of THC administered as any of the three treatments (*p* = 0.2204 for THC; *p* > 0.999 for the extract; and *p* = 0.2102 for the mix). In contrast, 10 nmoles of the CB2R antagonist AM630 continued to interfere with analgesia elicited by pure THC (*p* = 0.0046), the extract (*p* = 0.0349), and the mix (*p* = 0.0002). Dunnett’s *post hoc* tests further indicated that the response induced by this higher dose of pure THC was blocked by 25 nmoles of the TRPV1 channel blocker capsazepine (*p* < 0.001), while analgesia by both the extract (*p* = 0.9994) and the mix (*p* = 0.4931) was insensitive to this dose, requiring 50 nmoles of capsazepine to inhibit their respective responses (extract: *p* < 0.001; mix: *p* < 0.0001).

**FIGURE 7:**
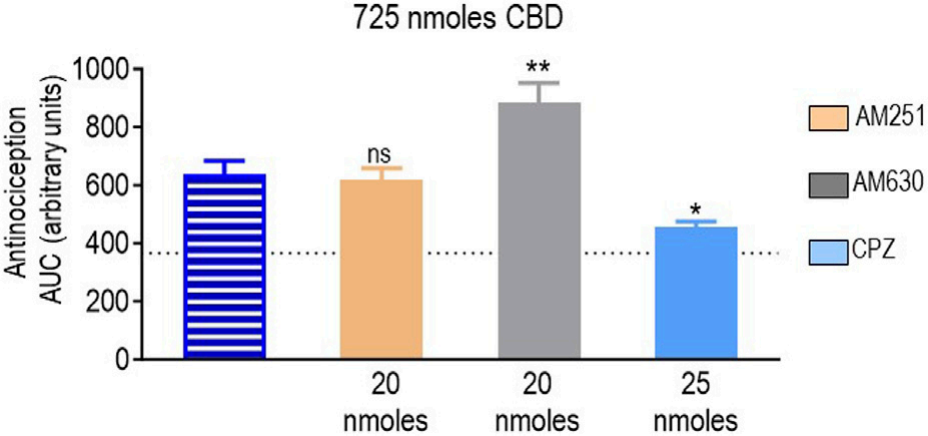
Effects of CB1R/CB2R antagonists and TRPV1 channel blockers on the anti-allodynic effects induced by CBD (725 nmoles). One week after induction of diabetes by STZ and 30 min before i.t. administration of CBD, rats were pre-injected (i.t.) with the selective CB1R antagonist AM251 (orange), the selective CB2R antagonist AM630 (gray), or the selective TRPV1 channel blocker capsazepine (sky blue bar), at the doses indicated in the figure. Histograms show AUC values of withdrawal thresholds integrated over 120 min and are expressed in arbitrary units. They correspond to the mean ± SEM, *n* = 6. Statistical comparisons were carried out using a one-way ANOVA followed by Dunnett’s multiple comparison test. *p < 0.05 and **p < 0.01, comparing antagonist vs. no antagonist pretreatment. Dotted lines indicate AUC values obtained in vehicle-injected animals, as detailed in the Materials and Methods section.

Finally, we determined how the different antagonists modified the antinociceptive actions induced by CBD at the only dose at which this cannabinoid-induced an effective response (725 nmoles; Figure 2D). A one-way ANOVA indicated an overall effect of pretreatment (*p* < 0.0001; Figure 7). A subsequent Dunnett’s test revealed that the CB1R antagonist AM251 did not show an effect (*p* = 0.9888), while the analgesia induced by CBD in the presence of the CB2R blocker AM630 was actually higher than when CBD was administered in its pure form (*p* = 0.0012). At 25 nmoles, the TRPV1 blocker capsazepine inhibited CBD response, leading to an increase in AUC values ∼25% higher than those observed in animals that had not received active treatment (*p* = 0.0182).

## Discussion

This study used a model of diabetic neuropathy to investigate how phytocannabinoid blending influences the antinociceptive responses of the major cannabinoids in the mix. We found that intrathecal administration of THC and CBD in their pure forms effectively engaged spinal/DRG targets of the endocannabinoid system to mitigate mechanical hypersensitivity in diabetic animals. Evidence of antinociception was also found when both products were co-administered either as a pure 1:1 mix or as an extract, but the levels of antinociception that were attained and the targets that were engaged by the same dose of THC were influenced by the different blends. These findings indicate that product composition influences pain mitigation by equivalent doses of THC and that the specificity of response is mediated, at least in part, by the distinct engagement of endocannabinoid targets.

### Antinociceptive responses elicited by THC and CBD

Intrathecal administration of pure THC or pure CBD both increased mechanical withdrawal thresholds in diabetic animals, although THC was more effective since it induced a higher maximal response at lower doses than CBD. These observations are congruent with previous results in models of diabetic neuropathy, where the administration of synthetic cannabinoids into the subarachnoid space was shown to effectively mitigate mechanical hyperalgesia (Ikeda et al., 2013; Goncalves et al., 2022). The observations in the present study are also consistent with the anti-allodynic actions reported for intrathecal administration of THC (Casey et al., 2022) or CBD (Xiong et al., 2012; Harding et al., 2023) in neuropathic pain models of nerve injury.

The antinociception elicited by THC was blocked by CB1R and CB2R antagonists. These observations are in keeping with the documented efficacy of THC as an agonist at both CB1R and CB2R (Felder et al., 1992; Soethoudt et al., 2017) and with previous observations indicating that spinal activation of both receptor subtypes by synthetic cannabinoids mitigates mechanical allodynia in diabetic animals (Ikeda et al., 2013; Goncalves et al., 2022). In contrast, in a model of nerve injury, THC analgesia was found to be CB1- but not CB2-dependent (Casey et al., 2022), suggesting that the targets mediating cannabinoid analgesic responses may vary depending on the underlying pathophysiology of chronic pain conditions. Unlike THC, we observed that analgesia mediated by CBD was unaffected by the CB1R antagonist AM251, but it was markedly enhanced by the CB2R blocker AM630. This latter observation indicates that in the model of diabetic neuropathy, CBD interaction with CB2R actively mitigated its own analgesic actions and is consistent with pharmacodynamic studies reporting potent negative allosteric modulation of CB2R by this cannabinoid (Thomas et al., 2007).

The fact that CBD analgesia was independent of CB1 and CB2 receptor activation adds to previous observations linking CBD to alternative targets. Indeed, intrathecal delivery of CBD in nerve injury models was shown to mitigate mechanical hypersensitivity via activation of either glycine (Xiong et al., 2012) or CaV3.2 channels (Harding et al., 2023). In the pain model we used here, most of the CBD response was inhibited by the TRPV1 channel blocker capsazepine (Kanai et al., 2006). The notion that TRPV1 channels may be the target of analgesic treatments is consistent with previous studies showing that intrathecal administration of TRPV1 agonists like the vanilloid capsaicin or the endogenous cannabinoid anandamide induced analgesic actions (Di Marzo et al., 2000; Starowicz et al., 2012). Since TRPV1 channels physiologically facilitate pain generation (Crosson et al., 2019), the analgesic actions of capsaicin and anandamide are thought to result from the initial activation of the channel followed by its desensitization (Di Marzo et al., 2000; Starowicz et al., 2012). Studies in cultured DRG neurons show that cannabinoids like CBD and THC can also activate and desensitize TRPV1 channels (Anand et al., 2021), indicating similar mechanistic underpinnings for TRPV1-mediated analgesia by these two major cannabinoids. Consistent with this mechanism, we observed that THC analgesia was sensitive not only to GPCR antagonists but also to capsazepine. Hence, while CBD analgesia primarily relied on TRPV1, THC could engage all three targets assessed. Its ability to produce analgesia via CB1R, CB2R, and TRPV1 may explain why antinociception by THC was evident at lower doses than CBD, whose analgesic actions relied on TRPV1 channels but were mitigated by its occupation of CB2R without the evident contribution of CB1R.

### Antinociceptive responses by a 1:1 mix of THC:CBD and by a balanced (type II) cannabis extract

The maximum withdrawal threshold attained following administration of 725 nmoles of the THC:CBD mix was significantly higher than the value attained by the same dose of CBD but lower than the maximal response induced by 250 nmoles of pure THC. From these observations, it is reasonable to conclude that when administered as a blend, CBD mitigated the antinociception induced by lower doses of THC. At 250 nmoles, analgesia by THC was largely dependent on CB1 and CB2 receptors with an additional contribution from TRPV1. Since CBD negatively modulates CB2R (Thomas et al., 2007), its pharmacodynamic profile may explain how this cannabinoid may have obliterated antinociception at lower THC-equivalent doses administered as a mix. Unlike these observations, a study that previously assessed the analgesic response to intrathecal administration of a 1:1 mix of THC and CBD found that this same combination synergistically alleviated mechanical allodynia in a model of nerve injury (Casey et al., 2022). Interestingly, in that study, CBD’s contribution to the analgesic response was mediated via the activation of CB2R (Casey et al., 2022), providing a plausible explanation for the divergent interactions observed for the THC:CBD mix in the two studies. However, the reason why CBD displayed opposing efficacies in both studies remains unclear and may depend on the different species used or on distinct pathogenic mechanisms being at play in the different neuropathic pain models that were used in the two studies.

When the THC:CBD mix became effectively analgesic at 480 nmoles, its effects were sensitive to CB2R and TRPV1 channel blockers. The documented ability of both cannabinoids to modulate the TRPV1 channels (Anand et al., 2021) may therefore account for this analgesic response. The fact that the CB2R was also involved suggests that, following initial inhibition by CBD, higher THC doses could overcome CBD’s inverse modulation of this receptor.

Balanced or type II extracts are characterized by a THC:CBD ratio that is near unity (Wallach, 2021). Such is the case for the extract we tested here, which contained THC:CBD at a 1:1.4 ratio together with minor cannabinoids such as cannabinol, cannabichromene, and cannabigerol (details in the Materials and Methods section). When evaluating the AUC responses, significant antinociception by the extract started at a dose of 95 nmol-equivalent THC and, in this sense, was more effective than the pure cannabinoid, whose analgesic effect appeared at 250 moles. Interestingly, the extract had a lower minimum effective dose and a higher maximum response than those observed for the blend of pure THC:CBD. Incongruence in analgesic responses induced by an extract and a mixture of similar THC:CBD ratios was previously described for a chemotype III extract in which CBD predominates over THC (Comelli et al., 2008). Differences were interpreted as an “entourage effect” provided by minor cannabinoids, terpenes, and flavonoids present in the extract, but a mechanism underlying their interaction was not established. In the aforementioned study, the treatment was administered systemically, so the suggested entourage effect between the blended products may have occurred at the pharmacodynamic or pharmacokinetic level. To the best of our knowledge, the present report is the first to compare analgesia induced by intrathecal administration of an extract and a corresponding blend, thus allowing us to study entourage effects at the level of pharmacodynamic targets without pharmacokinetic confounders. The use of antagonists indicated that CB1R, CB2R, and TRPV1 blockers interfered with antinociception induced by lower doses of the extract (250 nmoles), thus resembling THC more than the mix in this respect. As the concentration increased (480 nmol-equivalent THC), the pharmacodynamic profile of the extract resembled more that of the mix, as both blended products induced a comparable degree of antinociception by similarly engaging CB2R and TRPV1 channels. However, since 50 nmoles of capsazepine were required to block analgesia at higher doses of either product, we cannot rule out that both the extract and the mix may have engaged TRP channels other than TRPV1 or other targets.

Guidelines for the therapeutic use of cannabis products do not distinguish between products of different compositions, considering that a specified dose of THC or CBD will produce the expected degree of analgesia independent of other blend components. We tested this assumption by injecting pure and blended cannabinoids into the subarachnoid space. Although i.t. administration is uncommon in clinical practice, this approach provided construct validity for the verification of treatment equivalence. In fact, by avoiding pharmacokinetic interactions that modify the bioavailability of cannabinoids administered via the systemic route (Dumbraveanu et al., 2023; Zamarripa et al., 2023), i.t. injections allowed to control the concentration of different cannabinoids at endocannabinoid targets that are accessible from the subarachnoid space. By ensuring controlled target exposure, we revealed that blend components influenced THC antinociception via a pharmacodynamic mechanism involving the same endocannabinoid targets underlying pain mitigation by THC.

Finally, it is also important to consider some of the limitations of the study. First, the use of reflexive behaviors to assess antinociception has translational limitations since they do not represent the complexity of nociceptive perception in chronic pain conditions. Second, cannabinoids were dissolved in organic vehicles whose i.t. administration could induce motor artifacts that influence paw withdrawal thresholds. As described in the Materials and Methods section, when paw withdrawal thresholds were measured in animals that received different vehicles, the values obtained could not be distinguished from thresholds observed in diabetic animals that received no treatment at all, suggesting that this level of confounding was most likely not an issue.

In conclusion, this study provides evidence that CBD and minor cannabinoids found in cannabis products may influence THC antinociception via pharmacodynamic targets that mediate the desired analgesic actions of cannabinoids.

## Data Availability

The raw data supporting the conclusions of this article will be made available by the authors, without undue reservation.
