# Supplementary material for: A type II cannabis extract and a 1:1 blend of Δ(9)-tetrahydrocannabinol and cannabidiol display distinct antinociceptive profiles and engage different endocannabinoid targets when administered into the subarachnoid space
Source: Front Pharmacol. 2023 Sep 8;14:1235255. doi: 10.3389/fphar.2023.1235255 (PMC10514912; doi:10.3389/fphar.2023.1235255)
Supplement: Supplementary file 1 [file DataSheet1.docx]

Supplementary Material

A type II Cannabis extract and a 1:1 blend of Δ(9)-tetrahydrocannabinol and cannabidiol display distinct antinociceptive profiles and engage different endocannabinoid targets when administered into the subarachnoid space

Besma Benredjem*, Graciela Pineyro *

*** Correspondence:** Corresponding Author: [besma.benredjem@umontreal.ca](mailto:besma.benredjem@umontreal.ca), [graciela.pineyro.filpo@umontreal.ca](mailto:graciela.pineyro.filpo@umontreal.ca)

## Supplementary Figures


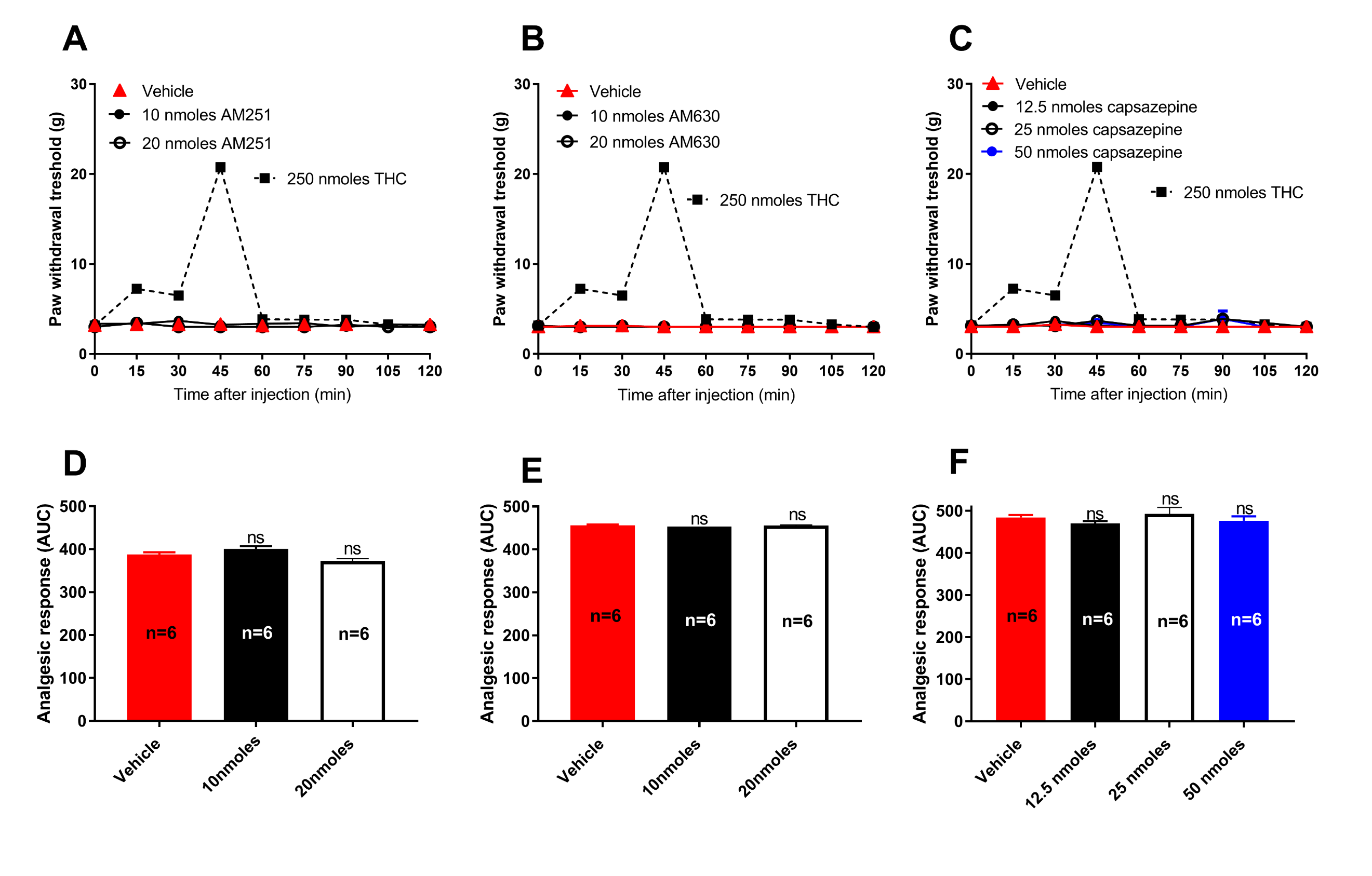


**Supplementary Figure 1. Antagonists for membrane targets of cannabinoids do not modify baseline withdrawal thresholds.** One week after administration of STZ, rats received i.t. injections of CB1R antagonist AM251 **(A, B)** CB2R antagonist AM630 **(C, D)** and TRPV1 antagonist capsazepine **(E, F)** at the indicated doses. The effect of THC (250 nmoles) is shown for comparison. Mechanical thresholds were assessed every 15 min after injection for a period of 120 min, thus allowing all responses to return to baseline. Histograms show AUC (mean ± SEM; n=6) corresponding to withdrawal thresholds integrated over 120 min and expressed in arbitrary units. One-way ANOVA followed by Sidak’s multiple comparisons test indicated no significant effect of **(B)** AM251 at 10 nmoles (p=0.2068) or 20 nmoles (p=0.1000); **(D)** AM630 at 10 nmoles (p=0.1393) or 20 nmoles (p=0.8466) and **(D)** capsazepine at 12.5 nmoles (p=0.6469) 25 nmoles (p=0.8946) or 50 nmoles (p=0.9100).


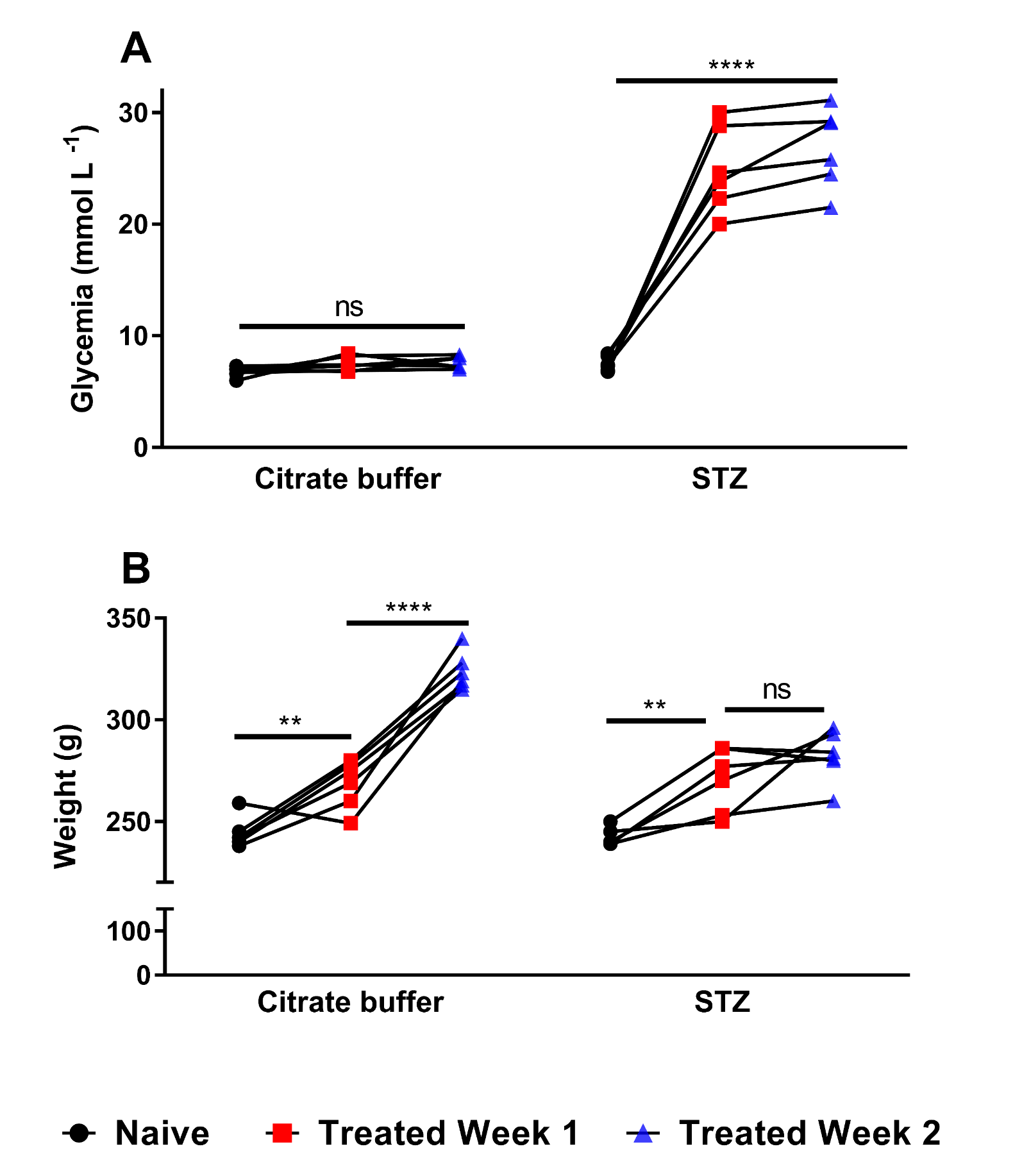


**Supplementary Figure 2. Evolution of non-fasting glycemia and body weight in type 1 diabetic rats.**  **(A)** Non-fasting glycemia was measured before (naïve; black circles, n=6), one week (red squares, n=6) and two weeks (blue triangles, n=6) after the injection of vehicle (Citrate buffer) or Streptozotocin (STZ), as indicated. Statistical significance of STZ-induced changes in blood glucose levels was evaluated using two-way ANOVA, which showed an effect of treatment *F* (1,30) = 291.10 (p < 0.0001) an effect of time *F* (2,30) = 77.41 (p < 0.0001) and an interaction *F* (2,30) = 64.98 (p < 0.0001). Tukey’s post-hoc test indicated a significant increase in glucose levels one and two weeks after STZ but not after citrate injection (p < 0.0001). Sidaks’s post-hoc test showed a significant increase in glucose levels = 64.98 (p < 0.0001), following STZ injection (p < 0.0001). **(B)** Body weight was evaluated before (naïve; black circles, n=6), one week (red squares, n=6) and two weeks (blue triangles, n=6) after vehicle (Citrate buffer) or Streptozotocin (STZ) treatments, as indicated. Statistical significance of STZ-induced changes in body weight was evaluated by two-way ANOVA, which showed an effect of treatment F (1, 30) = 13.23 (p = 0.001), an effect of time F (2, 30) = 86.68 (p < 0.0001) and an interaction F (2, 30) = 14.62 (p < 0.0001). At week one, Sidaks’s post-hoc test showed a significant increase in body weight following injection of both citrate buffer (p < 0.01) and STZ (p < 0.01). During the second week, the citrate buffer treatment group continued to show a significant increase in body weight compared to week 1 (p<0.0001), but this was not the case for the STZ treatment group (p=0.6547).

## Supplementary Tables

**Supplementary Table 1.** Vehicles used to assess analgesia induced by different doses of cannabinoids (*).

| **Vehicle used** | **Administered treatment** |
| --- | --- |
| **(1)** 5% DMSO, 5% Cremophor | WIN 55, 212-2 (0.2- 60 nmoles) |
| **(2)** 15% ethanol, 7.5% Cremophor | THC, CBD (95-480 nmoles); Extract (95-250 nmoles) |
| **(3)** 25% ethanol, 7.5% Cremophor | Extract (480-725 nmoles); THC, CBD (725 nmoles) |
| **(4)** 32 % ethanol, 7.5% Cremophor | THC:CBD (95-480 nmoles) |
| **(5)** 48 % ethanol, 7.5% Cremophor | THC:CBD (725 nmoles) |

*(*) Vehicles used contained the minimal amount of ethanol that allowed for product dilution at increasing concentrations.*

**Supplementary Table 2.** Vehicles used to assess analgesia induced by different cannabinoids had no effect on basal withdrawal thresholds (*).

|  | **Paw Withdrawal Threshold (g)** | |
| --- | --- | --- |
| **Treatments** | Mean | SEM |
| Streptozotocin (n=5) | 3.30 | 0.17 |
| **(1)** Vehicle (5% DMSO / 5% Cremophor) (n=5) | 3.66 | 0.26 |
| **(2)** Vehicle (11.6% Ethanol / 7.5% Cremophor) (n=5) | 3.26 | 0.11 |
| **(3)** Vehicle (24% Ethanol / 7.5% Cremophor) (n=5) | 3.37 | 0.18 |
| **(4)** Vehicle (31.7% Ethanol / 7.5% Cremophor) (n=5) | 3.26 | 0.11 |
| **(5)** Vehicle (48% Ethanol / 7.5% Cremophor) (n=5) | 3.40 | 0.17 |

*(*) Note that despite lack of effects on thresholds, vehicles used contained the minimal amount of ethanol that allowed for product dilution at increasing concentrations.*

Baseline measurement of mechanical sensitivity were taken 1 week following Streptozotocin administration (STZ, 50 mg·kg^−1^ per injection). Rats then received i.t. injections of indicated vehicle and mechanical thresholds were assessed every 15 min for 120 min. The mean withdrawal threshold (± SEM) over this time period was calculated for each vehicle and compared to the mean withdrawal threshold obtained in STZ rats that received no treatment. Statistical significance of vehicle-induced changes in mechanical allodynia as compared to non-injected STZ rats was evaluated by one-way ANOVA, which showed no effect of vehicle (p=0.5886).

Supplementary Table 3. Vehicles used to assess how CB1, CB2 and TRPV1 blockers interfered with analgesia induced by different cannabinoid treatments.

| **Vehicles used** | **Antagonist/cannabinoid combination tested** |
| --- | --- |
| **1)** VhAnt (2.5% DMF/5% Cremophor) +  VhTreat (5% DMSO/5% Cremophor) | AM630 (10nmoles) + WIN55212 (20 nmoles) |
| **2)** VhAnt (5% Ethanol/5% Cremophor) +  VhTreat (5% DMSO/5% Cremophor) | AM251(10 nmoles) + WIN55212 (20 nmoles)  CPZ (50 nmoles) + WIN55212 (20 nmoles) |
| **3)** VhAnt (5% DMF/5% Cremophor) +  VhTreat (25% Ethanol% /7.5%Cremophor) | AM630 (10 nmoles) + THC/Extract (250-480 nmoles)  AM630 (20 nmoles) + CBD (725 nmoles) |
| **4)** VhAnt (5% Ethanol/5% Cremophor) +  VhTreat (25% Ethanol% /7.5%Cremophor) | AM251 (10 nmoles) + THC/Extract (250-480 nmoles)  AM251 (20 nmoles) + CBD (725 nmoles)  CPZ (12.5-50 nmoles) + THC/Extract (250-480 nmoles)  CPZ (25-50 nmoles) + CBD (725 nmoles) |
| **5)** VhAnt (5% DMF/5% Cremophor) +  VhTreat (32% Ethanol% /7.5%Cremophor) | AM630 (10 nmoles) + THC-CBD (480 nmoles) |
| **6)** VhAnt (5% Ethanol/5% Cremophor) +  VhTreat (25% Ethanol% /7.5%Cremophor) | AM251 (10 nmoles) + THC-CBD (480 nmoles)  CPZ (25-50 nmoles) + THC-CBD (480 nmoles) |

**Supplementary Table 4.** Vehicles used in experiments assessing the effect of CB1, CB2 and TRPV1 blockers on cannabinoid analgesia had no effect on basal withdrawal thresholds (*).

|  | **Paw Withdrawal Threshold (g)** | |
| --- | --- | --- |
| **Treatments** | Mean | SEM |
| STZ (n=6) | 3.14 | 0.12 |
| **1)** Vh_Ant_ (2.5% DMF) + Vh_Treat_ (5% DMSO) (n=6) | 3.06 | 0.02 |
| **2)** Vh_Ant_ (5% Ethanol) + Vh_Treat_ (5% DMSO) (n=6) | 3.11 | 0.03 |
| **3)** Vh_Ant_ (5% DMF) + Vh_Treat_ (25 % Ethanol) (n=6) | 3.03 | 0.01 |
| **4)** Vh_Ant_ (5% Ethanol) + Vh_Treat_ (25 % Ethanol) (n=6) | 3.15 | 0.04 |
| **5)** Vh_Ant_ (5% DMF) + Vh_Treat_ (32 % Ethanol) (n=6) | 3.03 | 0.01 |
| **6)** Vh_Ant_ (5% Ethanol) + Vh_Treat_ (32 % Ethanol) (n=6) | 3.12 | 0.02 |

*(*) Note that despite lack of effects on thresholds, vehicles used contained the minimal amount of ethanol that allowed for product dilution at increasing concentrations.*

Baseline measurement of mechanical sensitivity were taken 1 week following Streptozotocin administration (STZ, 50 mg·kg^−1^ per injection, n=6). Rats then received i.t. injections of vehicle for different antagonists (Vh_Ant_), and 30 min later the vehicle for different active treatments (Vh_Treat_). Mechanical thresholds were assessed every 15 min for 120 min following administration of the vehicle for treatment (Vh_Treat_). The mean withdrawal threshold (± SEM) over this time period was calculated for each vehicle pair and compared to the mean withdrawal threshold obtained in STZ rats that received no treatment. Statistical significance of vehicle-induced changes in mechanical allodynia as compared to non-injected STZ rats was evaluated by one-way ANOVA, which showed no effect of vehicle (p=0.4093).
